# Pan-cancer patterns of DNA methylation

**DOI:** 10.1186/s13073-014-0066-6

**Published:** 2014-08-30

**Authors:** Tania Witte, Christoph Plass, Clarissa Gerhauser

**Affiliations:** Division of Epigenomics and Cancer Risk Factors, German Cancer Research Center (DKFZ), Im Neuenheimer Feld 280, 69120 Heid elberg, Germany

## Abstract

The comparison of DNA methylation patterns across cancer types (pan-cancer methylome analyses) has revealed distinct subgroups of tumors that share similar methylation patterns. Integration of these data with the wealth of information derived from cancer genome profiling studies performed by large international consortia has provided novel insights into the cellular aberrations that contribute to cancer development. There is evidence that genetic mutations in epigenetic regulators (such as DNMT3, IDH1/2 or H3.3) mediate or contribute to these patterns, although a unifying molecular mechanism underlying the global alterations of DNA methylation has largely been elusive. Knowledge gained from pan-cancer methylome analyses will aid the development of diagnostic and prognostic biomarkers, improve patient stratification and the discovery of novel druggable targets for therapy, and will generate hypotheses for innovative clinical trial designs based on methylation subgroups rather than on cancer subtypes. In this review, we discuss recent advances in the global profiling of tumor genomes for aberrant DNA methylation and the integration of these data with cancer genome profiling data, highlight potential mechanisms leading to different methylation subgroups, and show how this information can be used in basic research and for translational applications. A remaining challenge is to experimentally prove the functional link between observed pan-cancer methylation patterns, the associated genetic aberrations, and their relevance for the development of cancer.

## Introduction

Ongoing molecular characterizations of large cohorts of cancer patients using tumor samples from all major organs have made available a wealth of genomic, epigenomic, transcriptomic and proteomic data, enabling integrated analysis across different tumor types - so called pan-cancer analyses. These studies aim to identify genomic and epigenomic similarities and differences among distinct cancer types, independent of their tissue of origin [1]. The large number of available tumor sample datasets increases statistical power, allowing researchers to detect molecular aberrations that otherwise would have been missed. From these integrated analyses, mutational landscapes are emerging that have revealed novel oncogenic signatures and cancer driver mutations [2-4].

Cancer is no longer seen as solely a genetic disease; epigenetic alterations are now being taken into account as additional layers in the regulation of gene expression. Epigenetic modifications, including DNA methylation, non-coding RNAs, histone modifications and nucleosome positioning, modify chromatin structure and hence gene transcription. These mechanisms act coordinately to form an epigenetic landscape regulated by various enzymes, either establishing (writers), interpreting (readers), modifying (editors) or removing (erasers) epigenetic marks (reviewed in [5]).

DNA methylation is by far the best characterized epigenetic modification and is involved in the regulation of gene expression, genome stability and developmental processes (reviewed in [6]). High-throughput techniques, including array and sequencing-based technologies, now provide genome-scale DNA methylation maps (also called methylomes), which have confirmed aberrant methylation as a hallmark of all cancer types and are used to identify novel methylation-based cancer biomarkers.

Multidisciplinary international consortia such as The Cancer Genome Atlas (TCGA) or the International Cancer Genome Consortium (ICGC) have produced methylomes for thousands of samples from at least 15 cancer types (Box 1). Integrative data analyses have revealed that methylomes in subgroups within one tumor type might differ more than between distinct cancer types. Even within the same tumor, regional differences in DNA methylation alterations have been identified, associated with intrinsic tumor heterogeneity [7].

The TCGA Pan-Cancer project was launched in 2012 with the goal of collecting, analyzing and interpreting data across distinct tumor types and of making these resources publically available [2]. One of the aims of this project is to define pan-cancer methylation patterns and to integrate them with genomic, transcriptomic and proteomic data. A remarkable initial finding was that tumor samples cluster largely according to their tissue of origin [1]. Analyses of single tumor entities revealed that colorectal, gastric and endometrial cancers have similar highly methylated subgroups that are associated with tumors with microsatellite instability and hypermethylation of the *MLH1* promoter. Subtypes of breast, serous endometrial, high-grade serous ovarian, colorectal and gastric carcinomas are associated with high chromosomal instability as well as with recurrent *TP53* mutations and share patterns of low methylation. Moreover, emerging evidence shows that cancer genomes exhibit frequent mutations in epigenetic regulators, suggesting a close interplay between epigenomic and genomic events (reviewed in [8]). Identifying commonalities between tumor entities might help to identify therapeutic regimens that are in place for one tumor type as being of use for another, less well characterized one, and will allow better patient stratification [1]. Deciphering the mechanisms underlying methylation patterns will facilitate the identification of novel therapeutic targets.

In this review, we aim to highlight recent findings from genome-wide DNA methylation profiling studies. We describe DNA methylation subgroups in 11 distinct tumor entities and analyses across cancer types, and discuss the potential mechanisms underlying the different methylation subgroups. We also explore the potential use of DNA methylation as a biomarker for diagnostic, prognostic and treatment response, and as a target for epigenetic therapy.

## Definition and function of DNA methylation

DNA methylation usually occurs at cytosine-guanine (CpG) dinucleotides, where DNA methyltransferases (DNMTs) catalyze the transfer of a methyl group to position 5 of a cytosine, generating 5-methylcytosine (5mC). DNMT1 maintains the patterns of DNA methylation after cell division using hemi-methylated DNA as a template [9], while the *de novo* methyltransferases DNMT3A and DNMT3B establish cytosine methylation during early development [10]. For a long time, it was believed that methyl groups are only removed passively after cell replication. However, active mechanisms of DNA demethylation were recently identified. For instance, DNA repair pathways have an essential role in the active removal of 5mC, involving proteins such as GADD45 (reviewed in [11]). Another mechanism implicates the ten-eleven translocation (TET) family of proteins, which catalyze the hydroxylation of 5mC to 5-hydroxymethylcytosine (5hmC) [12]. Subsequent studies showed that 5hmC can be further converted to 5-formylcytosine and/or 5-carboxylcytosine, which can then be excised by thymine-DNA glycosylase [13].

The location and distribution of 5mCs across the genome have important implications for understanding the roles of DNA methylation [6]. In mammalian genomes CpGs are unevenly distributed: they are depleted on a global scale but enriched at short CpG-rich DNA stretches known as CpG islands (CGIs), which are preferentially located at transcription start sites of gene promoters (reviewed in [14]). In normal cells, cytosines within CGIs are generally protected from DNA methylation, in contrast to the vast majority of CpGs, which are usually methylated (that is, at non-coding regions and repetitive elements) [15]. Flanking regions of CGIs (±2 kilobases), referred to as CGI shores, show tissue-specific DNA methylation and are associated with gene silencing [16].

**Figure 1:**
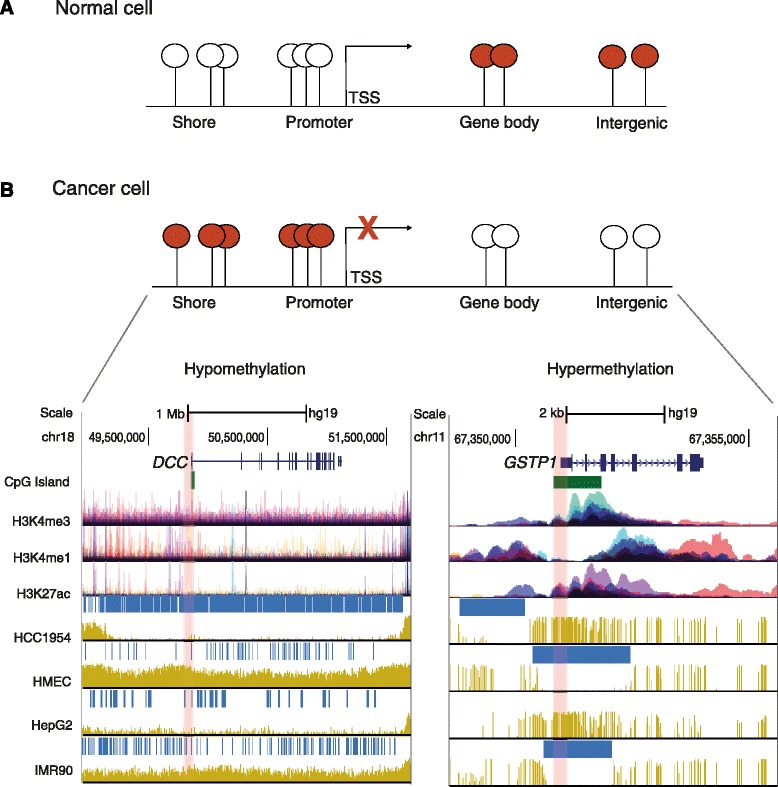
**DNA methylation patterns in normal and cancer cells. (A)** In normal cells, most CpGs located outside of promoters in gene bodies and intergenic regions are methylated (red circles), whereas promoter-associated CpG islands are protected from DNA methylation (white circles). **(B)** In cancer cells, a global or localized loss of 5-methylcytosine occurs at gene bodies and intergenic regions, whereas CpG-rich regions like promoters are usually heavily methylated, which might lead to transcriptional repression. Regions of intermediate CpG levels such as shores are associated with tissue-specific methylation. Global loss (left plot) and focal gain (right plot) of DNA methylation are depicted as tracks of the University of California Santa Cruz genome browser [118] using whole-genome bisulfite sequencing data for normal and cancer cell lines. Tracks for CpG islands and selected histone modifications, including H3K4me3, which is associated with transcriptionally active promoters, and H3K4me1 and H3K27ac as markers for enhancers, are illustrated below the gene track. Each color of the histone tracks represents an individual ENCODE cell line. The deleted in colon cancer gene (*DCC*) was taken as an exemplary locus for which long-range hypomethylation regions (horizontal blue bars) are observed in the breast cancer cell line HCC1954 and in the liver carcinoma cell line HepG2, but not in normal mammary epithelial cells (HMEC) or the myofibroblast cell line IMR90. The glutathione *S-*transferase P1 gene (*GTSP1*) represents an example of promoter hypermethylation (highlighted in red) in cancer cell lines compared to normal cells. TSS, transcription start site.

The patterns of DNA methylation observed in normal genomes change during tumorigenesis. The first epigenetic alteration reported in cancer cells was a widespread loss of 5mC [17], which has been recently confirmed in single-base-resolution methylomes of colorectal cancer, chronic lymphocytic leukemia (CLL) and medulloblastoma [18-20]. Loss of DNA methylation occurs mainly at repetitive sequences, centromeric DNA and gene bodies, leading to genomic instability, reactivation of transposable elements or loss of imprinting, which ultimately contribute to tumor initiation or progression [21]. Hypomethylation can also lead to transcriptional activation of normally silenced genes such as oncogenes (reviewed in [22]). Additionally, whole-genome bisulfite sequencing (WGBS) analyses have shown that global hypomethylation usually coincides with large partially methylated domains (PMDs) that are associated with late replication lamina-associated domains and might lead to long-range epigenetic silencing through repressive chromatin domain formation [23,24]. Recent studies have also revealed that hypomethylation occurs at more localized regions, termed DNA methylation valleys (DMVs), which are enriched for developmental genes and may regulate tissue-specific expression [20,25]. Global or localized DNA hypomethylation in cancer cells is often accompanied by focal hypermethylation of CGIs (Figure 1), which contributes to carcinogenesis by transcriptional silencing of genes including tumor suppressor genes (TSGs) [26].

## DNA methylation subgroups according to tumor types

It has long been thought that each tumor type has a characteristic DNA methylation pattern. For example, a specific pattern of high methylation at CGIs, defined as the CpG island methylator phenotype (CIMP), was first discovered in colorectal cancer [27], even before the omics era. Now, genome-wide sequencing projects have confirmed the existence of this and additional DNA methylation subgroups in multiple cancer types. The question remains as to what extent these DNA methylation patterns are unique for a specific tumor type or comparable across different types of cancers. The comprehensive molecular catalogs generated by the TCGA might help to shed light on this (summarized in Table 2).

**Table 1:** **International Cancer Genome Consortium projects with methylomes generated by Infinium BeadChips**

**Tumor type**	**Project and country identification**	**Number of methylomes**
Breast	BRCA-US	971
Ovary	OV-US	572
Kidney	KIRC-US	491
Head and neck	THCA-US	488
Uterus	UCEC-US	481
Lung	LUAD-US	460
Colorectal	COAD-US	414
Lung	LUSC-US	410
Head and neck	HNSC-US	407
Brain	GBM-US	393
Skin	SKCM-US	338
Stomach	STAD-US	328
Brain	LGG-US	293
Bladder	BLCA-US	198
Prostate	PRAD-US	196
Blood	LAML-US	194
Pancreas	PACA-AU	167
Blood	CLLE-ES	159
Colorectal	READ-US	150
Liver	LIHC-US	149
Kidney	KIRP-US	142
Cervix	CESC-US	127
Brain	PBCA-DE	115
Ovary	OV-AU	93
Pancreas	PAAD-US	72
Pancreas	PAEN-AU	23

Modified from the International Cancer Genome Consortium data portal [104]. AU, Australia; DE, Germany; ES, Spain; US, United States.

**Table 2:** **Pan-cancer patterns of DNA methylation**

**Tumor type (number of methylation groups)**	**Methylation subgroup**	**Genomic aberrations**	Methylation pattern*	**Comments**	**References**
AML	High	*IDH1/2* or *TET2* mutations	A	Associated with patients presenting with an intermediate-risk karyotype	[43,51,107]
				Co-occurrence of *IDH1/2* and *NPM1* mutations is associated with good clinical outcome	
Bladder urothelial (3)	High	*RB1* mutations		Smoking-pack years as predictor of CIMP phenotype Frequent mutations in chromatin regulators such as *MLL2*, *ARID1A*, *KDM6A*, and *EP300* ^†^ Mutations in chromatin regulators were more frequent than in any other TCGA tumor	[35]
	Low	↑ *TP53* mutations	B		
Breast (5)	B-CIMP	↓ mutation rate		Luminal ER/PR-positive tumors	[31,32]
				Low metastatic risk and better clinical outcome	
				Enriched for genes targeted by the PRC2 (e.g. *SUZ12* and *EZH2*)	
	B-CIMP-negative	↑ *TP53* mutations	B	Basal-like tumors (ER/PR-negative)	
				High metastatic risk and poor clinical outcome	
Cholangiocarcinoma	High	*IDH1* and/or *IDH2* mutations	A	Longer survival	[47]
Chondrosarcoma	High	*IDH1* and/or *IDH2* mutations	A		[46,64]
Colorectal (4)	CIMP-H	*MLH1* hypermethylation	C	MSI	[29,108]
				Right/ascending colonic region	
		↑ mutation rate			
		↑ *BRAF* ^*V600E*^ mutation			
				Good prognosis	
		↑ *BRAF* ^*V600E*^ mutation			
	CIMP-L	*KRAS* mutations		CIN (non-MSI)	
				Poor prognosis	
	Two non-CIMP	↑ *TP53* mutations	B	Anatomic origins distinct from CIMP groups	
		↑ SCNAs			
Endometrial (4)	High	*MLH1* hypermethylation	C	MSI	[33]
				*ARID5B* mutations	
		↑ mutation rate			
	Low	↑ *TP53* mutations	B	Serous-like tumors	
		↑ SCNAs		Poor prognosis	
	Two non-methylated	↑ *POLE* mutations		Endometrioid tumors	
		↑ SCNAs		*ARID1A* and *PTEN* mutations were present in all groups without high *TP53* mutations	
Gastric (4)	EBV-CIMP	↑ *PIK3CA*, *ARID1A* and *BCOR* mutations		EBV-positive tumors Highest frequency of hypermethylation events among TCGA tumors	[30]
		*CDKN2A* hypermethylation			
		Amplifications of *JAK2*, *CD274* and *PDCD1LG2*			
	Gastric CIMP	*MLH1* silencing	C	MSI	
		↑ mutation rate			
	Cluster 3 – low	*RHOA* and *CDH1* mutations		Enriched for the diffuse histological variant	
		Genomically stable		Also fusions involving RHO-family GTPase-activating proteins	
	Cluster 4 – low	↑ *TP53* mutation	B	CIN	
		Focal amplifications of receptor tyrosine kinases			
Glioblastoma (6)	G-CIMP	*IDH1* mutations	A	Secondary tumors with proneural expression	[41,42,48]
		*ATRX* mutations			
		*MYC* mutations and amplifications			
				Younger age at diagnosis	
				Better survival rates	
	G-CIMP negative proneural	No *IDH1* mutations		Relative hypomethylation	
		*PDGFRA* amplifications		Proneural subtype cases without *IDH1* mutations	
Pediatric glioblastoma (6)	Global loss of methylation at non-promoter regions	*H3F3A* mutations		*H3F3A* mutations are mutually exclusive with *IDH1* mutations and are associated with *TP53* mutations and alternative lengthening of telomeres (ALT)	[49,109]
Renal cell carcinoma	Global loss of methylation	*SETD2* mutations		*VHL* hypermethylation in about 7 % of the tumors^†^	[36]
				Loss of methylation at non-promoter regions	
				One of the tumor types with the lowest frequency of DNA methylation events	
Lung ADCA (3)	CIMP-high	*CDKN2A* hypermethylation		Associated either with ↑ ploidy, ↑ mutation and the PI subtype or with ↓ ploidy, ↓ mutation rate and the TRU subtype	[39]
		*MYC* overexpression			
				Mutations in chromatin modifiers such as *SETD2*, *ARID1A*, *SMARCA4* ^†^	
Lung SQCC (4)	High	*CDKN2A* inactivation		Classical expression subtype	[38]
		*NFE2L2*, *KEAP1*, *PTEN* mutations			
				Chromosomal instability	
		↑ SCNAs			
	Low			Primitive expression subtype	
Serous ovarian (4)	High	Germline and somatic *BRCA1* mutations		More differentiated tumors	[34]
				Better survival	
	Low	↑ *TP53* mutation	B	*TP53* mutations occur in 90 % of the tumors and are not exclusive for the low methylation group	
		↑ SCNAs			
		*BRAC1* hypermethylation			
				Poor clinical outcome	

*Methylation patterns A, B and C indicate common genetic and epigenetic aberrations across different tumors. ^†^These molecular aberrations were not necessarily associated with a specific methylation subgroup. ADCA, adenocarcinoma; AML, acute myeloid leukemia; CIMP, CpG island methylator phenotype; CIN, chromosomal instability; EBV, Epstein-Barr virus; ER, estrogen receptor; MSI, microsatellite instability; PI, proximal inflammatory; PR, progesterone receptor; PRC, polycomb repressor complex; SCNAs, somatic copy-number alterations; SQCC, squamous cell carcinoma; TCGA, The Cancer Genome Atlas; TRU, terminal respiratory unit.

However, a caveat should be noted: the methylation data underlying these reports were derived from 27 k and 450 k Illumina platforms. Only CpG sites covered on both platforms were considered and filtered for sites overlapping with single-nucleotide polymorphisms, resulting in around 10,000 eligible CpGs. From these, the most variable CpG sites were used for cluster analyses. The interpretation of these datasets is to a certain extent biased, as 27 k arrays mainly cover sites located within CGIs, while information on additional regulatory regions (for example, shores, intra- and intergenic enhancers) is missing. Also, information on larger genomic domains such as PMDs and DMVs cannot be determined from these datasets.

### Colorectal and gastric cancer 

According to the degree of methylation, colorectal cancer is currently divided into four DNA-methylation subgroups with specific genetic and clinical features [28,29]; that is, CIMP high (CIMP-H), CIMP low (CIMP-L) and two non-CIMP subgroups. CIMP-H is associated with hypermethylation of the repair gene *MLH1*, the activating *BRAF*^*V600E*^ mutation and microsatellite instability (MSI). Tumors in this subgroup are often derived from the right/ascending colon, show high mutation rates (hypermutation) and low somatic copy-number alterations (SCNAs). The molecular mechanisms underlying these relationships need more investigation. CIMP-L is associated with tumors enriched for *KRAS* mutations and chromosomal instability (non-MSI). The non-CIMP subgroups, corresponding to the majority of colorectal tumors, do not show specific mutations, but are enriched for SCNAs and originate from distinct anatomical sites compared with the CIMP groups.

Epstein-Barr virus (EBV)-positive gastric tumors display an extreme EBV-CIMP profile [30], with hypermethylation of *CDKN2A* but not of *MLH1*. This phenotype has the highest frequency of DNA hypermethylation when compared with other cancer types reported by TCGA [30]. In contrast, gastric CIMP tumors showed hypermutation, MSI and epigenetic silencing of *MLH1*.

### Breast, endometrial and ovarian carcinomas 

A breast CpG island methylator phenotype (B-CIMP) was first reported in 2011 [31]. B-CIMP is enriched in estrogen and progesterone receptor (ER/PR)-positive tumors and is associated with good survival rates and low metastatic risk. It is characterized by high methylation of genes targeted by the polycomb repressor complex 2 (PRC2), including *SUZ12* and *EZH2* [31]. In contrast, the B-CIMP-negative group shows high metastatic risk and poor clinical outcome. TCGA analyses confirmed these findings, although they defined five distinct DNA methylation subgroups. The high methylation group overlapped with luminal B tumors (ER/PR-positive) and had a low rate of mutations. Conversely, the methylation-low group had a high *TP53* mutation rate and was enriched in basal-like tumors (ER/PR-negative) [32].

In endometrial carcinomas, TCGA identified four DNA methylation subtypes. Similar to colorectal cancer, the high methylator phenotype was mainly composed of hypermutated MSI tumors showing extensive *MLH1* promoter hypermethylation and an under-representation of *TP53* mutations [33].

Four DNA methylation clusters were defined for serous ovarian cancer. This cancer type has a 90 % prevalence of *TP53* mutations. TCGA identified a methylation-high group enriched for highly differentiated tumors with germline *BRCA1* mutations. *BRCA1* mutations were mutually exclusive with *BRCA1* hypermethylation, which is characteristic of methylation-low tumors with high SCNAs. Survival analysis showed that cases with hypermethylated *BRCA1* had a poorer clinical outcome compared to tumors with *BRCA1/2* mutations [34].

### Bladder urothelial and kidney renal clear cell carcinomas 

Bladder urothelial carcinomas were divided into three DNA methylation subgroups; one of these groups had a CIMP-like hypermethylation profile and was enriched for tumors with *RB1* mutations. Similar to the low methylation groups in breast, endometrial, gastric and colorectal tumors, the methylation-low group had the highest percentage of *TP53* mutations, suggesting a common molecular mechanism of epigenetic regulation. Interestingly, chromatin regulators such as the histone methyltransferase *MLL2*, the chromatin remodeling gene *ARID1A*, the histone demethylase *KDM6A* and the histone acetyltransferase *EP300* were frequently mutated in this cancer type [35].

For renal clear cell carcinoma, the most common type of kidney cancer, TCGA identified epigenetic silencing of the tumor suppressor *VHL* in about 7 % of the tumors, which was mutually exclusive with *VHL* mutations. Increased promoter methylation was linked to tumors with a higher grade and stage. Tumors with a widespread loss of DNA methylation were associated with mutations of the H3K36 methyltransferase *SETD2*, in contrast to methylation-low subgroups in other cancer types [36].

### Lung adenocarcinoma and squamous cell carcinoma 

Non-small-cell lung carcinoma (NSCLC), the most common type of lung cancer, is divided into three subtypes: adenocarcinoma, squamous cell carcinoma (SQCC), and large cell carcinoma [37]. Methylation analysis of SQCC identified four groups with distinct DNA methylation patterns. The methylation-high group overlapped with tumors from the so-called classical subtype, which are characterized by chromosomal instability. Moreover, the TSG *CDKN2A* was inactivated in 72 % of cases, 21 % of which were due to epigenetic silencing [38].

Recent results for adenocarcinoma revealed three different methylation subgroups: CIMP-H, a subgroup with intermediate methylation levels, and CIMP-L. Remarkably, these methylation subgroups were not specifically related to genomic, transcriptomic or histopathological subtypes. CIMP-H subtypes were either associated with tumors with high ploidy and a high mutation rate and were classified as proximal inflammatory (previously known as squamoid), or were associated with tumors presenting with low ploidy and a low mutation rate and were classified as terminal respiratory unit (formerly bronchioid). Moreover, an association between tumors enriched for *SETD2* and *CDKN2A* methylation was found, suggesting an interaction between *SETD2* mutations and altered chromatin structure for these tumors [39].

### Glioblastoma 

Aberrant DNA methylation has been widely described for glioblastoma multiforme (GBM) – the most common adult brain tumor. In 2008, TCGA chose GBM as the first cancer to be comprehensively characterized, revealing an important association between *MGMT* methylation, mutations in mismatch repair genes and response to therapy [40]. Subsequently, TCGA identified three DNA methylation groups, one of which showed hypermethylation at a large number of loci and was termed G-CIMP [41]. This group was enriched in secondary tumors with proneural expression and somatic mutations of the isocitrate dehydrogenase 1 (*IDH1*) gene [42]. This gain-of-function mutation results in increased catalysis of α-ketoglutarate to d-2-hydroxyglutarate (2-HG), which inhibits the activity of TET and KDM proteins, affecting chromatin remodeling and leading to an increase in DNA methylation. *IDH1/2* mutations are also common in hematopoietic malignancies, including acute myeloid leukemia (AML) [43], myelodysplastic syndromes (MDS), myeloproliferative neoplasms [44] and T-cell lymphomas [45], as well as in solid tumors such as chondrosarcoma [46] and cholangiocarcinoma [47].

The G-CIMP group is associated with better survival compared with G-CIMP-negative tumors. The survival advantage of G-CIMP tumors was confirmed by a follow-up TCGA study characterizing more than 500 GBM tumors [48]. In this study, six DNA methylation clusters, including the G-CIMP subgroup, were identified. Additionally, the G-CIMP phenotype was associated with a younger age at diagnosis, enrichment for mutations in the chromatin remodeling gene *ATRX*, and *MYC* alterations.

The landscape of DNA methylation and genomic aberrations in pediatric GBM varies. Instead of having a hypermethylator phenotype, these tumors show a global loss of 5mC, which is mainly associated with extensive changes in histone modifications caused by mutations in *H3F3A* (reviewed in [8]). This was defined by Sturm *et al.*, who found six epigenetic subgroups harboring specific mutations, SCNAs and transcriptome patterns [49]. Two methylation subgroups specifically correlated with hotspot mutations in *H3F3A*, namely at K27 and G34, and were associated with a younger age at diagnosis. Strikingly, the G34 tumors showed a global loss of methylation occurring mainly at chromosome ends. The presence of *IDH1* mutations was mutually exclusive with *H3F3A* mutations.

### Acute myeloid leukemia 

AML is a highly heterogeneous myeloid disorder and the most common acute leukemia in adults. AML patients from the normal or intermediate cytogenetic risk category frequently have mutations in epigenetic regulators such as *IDH1/2*, *DNMT3* and *TET* enzymes (reviewed in [50]). Similar to GBM, AML with a DNA hypermethylation phenotype is associated with *IDH1/2* mutations [43]. These mutations are mutually exclusive with mutations in the demethylating enzyme *TET2*, suggesting a complementary role. It might be that DNA methylation is a consequence of mutant *IDH* expression and that this phenotype contributes to AML development. The association of *IDH1/2* mutations with the hypermethylation phenotype in AML was confirmed by a recent TCGA study. Gain of DNA methylation was mainly observed at CpG-sparse regions of the genome. Other subtypes of tumors were associated with a substantial loss of DNA methylation and with the presence of *MLL* fusion genes or co-occurring mutations in *NPM1*, *DNMT3A* or *FLT3* [51].

### Potential mechanisms leading to DNA methylation subgroups 

The observation that many tumor types carry numerous mutations in enzymes regulating epigenetic patterns suggests that these defects contribute to the global alterations seen in cancer genomes [5,8]. However, despite this expected molecular link, there are currently only reports associating methylome subgroups with gene mutations [29,49], rather than detailed molecular studies. Exceptions are studies on the histone H3.3 mutation H3F3A(K27M), which inactivates *EZH2* in the PRC2 complex [52-54]. In addition, introduction of an *IDH1* mutant, R132H, into astrocytes induces a specific methylome pattern [55]. Mutations in *IDH1/2* cause accumulation of the oncometabolite 2-HG, which disturbs the DNA demethylation process, causing hypermethylation [43].

Epigenetic subgroups might also represent preexisting epigenetic states. For example, PRC2 target genes are commonly hypermethylated in cancer, and *EZH2* is up-regulated in various cancer subtypes. These changes were associated with gene amplifications, and alterations in the regulation of gene expression by noncoding RNAs and mutations (reviewed in [56]). Apart from mutations affecting epigenetic modifiers, other genes are certainly also affected. Colorectal CIMP is tightly associated with *BRAF* mutations, although it appears that these mutations do not drive the hypermethylation phenotype [28]. Methylation subgroups might reflect the survival advantage of cell populations that have acquired early defects in DNA repair genes (for example, *MLH1*, *MGMT* and *BRCA1*). Distinct methylation clusters might also represent a common cell type of origin. As an example, the basal breast cancer subgroup shares characteristics of low methylation, high *TP53* mutations and high chromosomal instability with serous endometrial and serous ovarian cancer subgroups [33]. Different epigenetic subgroups have been suggested to represent differences in tumor etiology induced by environmental factors, such as recently shown for EBV in gastric cancer [30].

Again, the question of whether there is a causal relationship between epigenetic changes and cancer or whether these associations represent changes in the methylome that are non-functional events and thus do not contribute to the carcinogenic process (passengers) rather than methylation events that drive the carcinogenic process (drivers) remains open. However, there are some general observations that extend across studies. First, mutations in epigenetic enzymes such as *IDH1/2* are causally linked to the pathogenesis of subtypes of GBM and AML, as well as to the formation of CIMP. Second, mutations in the gene *H3F3A* encoding the histone variant H3.3 are associated with global loss of methylation, especially in sub-telomeric regions, and with the alternative lengthening of telomeres phenotype that is characteristic of a fraction of cancer cells, for example in pediatric GBM. Third, mutations in chromatin regulatory factors such as *SETD2*, *ARID1*, S*MARCA4*, *KDM6A*, *EP300* and *MLL* are emerging in various cancer types [57] but, so far, only a few have been linked to altered methylome patterns. Many of these factors act in protein complexes, indicating that mutations in any of these could disrupt the function of the complex. Fourth, current cancer epigenome research points to the fact that methylation of polycomb group targets (PCGTs) is detectable even in pre-neoplastic lesions and could represent a risk factor for neoplastic transformation [58]. Fifth, recent reports have described particular methylation patterns related to infectious agents such as EBV or human papilloma virus (HPV), which can initiate carcinogenesis [30,59]; whether these methylation alterations are primarily useful biomarkers for patient stratification or whether there is a causal relationship to carcinogenesis has yet to be demonstrated. Last, similarities in methylation patterns across tumor types could indicate the accumulation of as yet unidentified, low frequency molecular aberrations that lead to a common phenotype and contribute to cancer development. Future research will have to address these points to draw clear conclusions.

## Methylome analyses across different cancer types

The genome-wide methylation profiles generated by TCGA and others have shown that aberrant methylomes are a hallmark of cancer, and are useful for classifying tumor subgroups as well as for identifying novel clinical biomarkers. Currently, efforts are being made to integrate different methylomes and to determine common and tissue-specific DNA methylation patterns across multiple tumor entities (pan-cancer). These integrative analyses might also help to distinguish the driver methylation events (that contribute to the carcinogenic process) from the passenger methylation events (which do not contribute to the carcinogenic process).

**Figure 2:**
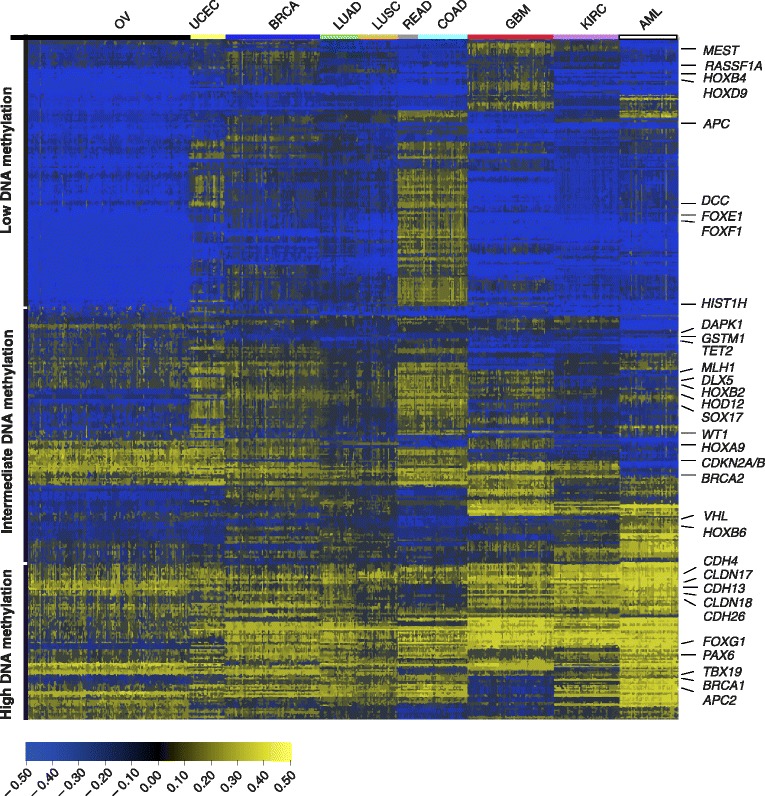
**Pan-cancer methylome representation for ten cancer cohorts from The Cancer Genome Atlas.** The Cancer Genome Atlas PANCAN12 DNA methylation data, representing 24,980 CpG sites acquired from the 27 k Illumina platform and corresponding to 2,224 tumor samples, were downloaded from the University of California Santa Cruz Cancer Genomics Browser [119]. CpG sites located on chromosome X and Y were removed, as well as the ones associated with single-nucleotide polymorphisms (*n* = 2,750). DNA methylation data for ten tumor entities - OV (*n* = 600), UCEC (*n* = 117), BRCA (*n* = 315), LUAD (*n* = 126), LUSC (*n* = 133), READ (*n* = 67), COAD (*n* = 166), GBM (*n* = 287), KIRC (*n* = 219) and AML (*n* = 194) - are included in the PANCAN12 dataset. For each of the tumor entities, color-coded on the top of the graph, the 500 most variable CpGs of the remaining 21,844 data points were selected. From the overlap, Qlucore Omics Explorer 3.0 software was used to select the 1,430 most variable CpGs, which were then hierarchically clustered as a heat map. Beta values are offset by −0.5 to shift the whole dataset to values between −0.5 (in dark blue) and 0.5 (in yellow) for improved graphical display [119]. DNA methylation patterns show relatively high homogeneity within tumor entities. We do not observe a common CpG island methylator phenotype-like group across several tumor types, suggesting that the ‘tissue of origin’ methylation signature is a strong decisive factor for the pattern. Colorectal cancer shows the highest overall methylation, whereas kidney cancer is characterized by low variance of methylation. The methylation patterns of ovarian, endometrial and breast cancer display a similar distribution of high and low methylation. CpG sites fall into high and intermediate DNA methylation clusters, covering all tumors entities, and a low methylation cluster with genes methylated in glioblastoma multiforme (GBM) or colorectal tumors and unmethylated in ovarian cancer. Unexpectedly, the high methylation cluster shows enrichment for membrane-associated genes including claudins (CLDN) and cadherins (CDH), while polycomb repressor complex PRC2 target genes are highly enriched in the intermediate and low methylation clusters. Some of these genes, as well as a selection of differentially methylated genes mentioned in the text such as *MLH1*, *APC*, *BRCA1/2* and *VHL*, are indicated on the right side of the graph. For abbreviations of the tumor entities see Table 1.

In 2013, TCGA published the first integrative analysis of genomic data across 12 cancer types. In this study, SCNAs, somatic mutations and DNA methylation were integrated, although methylation changes were limited to a selection of 13 epigenetically silenced genes. From these genes, *MGMT*, *GSTP1*, *MLH1* and *CDKN2A* were found to be aberrantly methylated in a large number of samples in different types of tumors. Hypermethylation of *MLH1* was associated with the so-called ‘M class’, characterized by recurrent mutations, whereas *BRCA1* hypermethylation correlated with the ‘C class’ of tumors enriched for SCNAs [3]. These findings confirm the previous TCGA reports for single tumor entities. However, by using this selected panel of genes, the results of this investigation might not reflect the actual similarities and differences in DNA methylation patterns across distinct tumor types, as for example shown in Figure 2.

By combining the methylomes of ten distinct tumor entities, Kim *et al.* found that aberrant DNA methylation affects similar biological pathways across the cancer types analyzed [60]. Over 50 % of the hypermethylation events were involved in early development and morphogenesis, including neurogenesis and embryonic development, whereas the remaining hypermethylation changes were related to transcription factor activity. A significant overlap between those pathways and PCGT genes was observed. Among the pan-cancer hypermethylated genes targeted by PRC2 were several members of the *HOX* family as well as the TSG *CDKN2A*. This finding is in agreement with previous studies reporting that methylation of PCGT genes is frequent in distinct cancer types (reviewed in [61]).

The integration of genome-wide DNA methylation data across four different gynecological tumors, namely breast, ovarian, endometrial and cervical carcinomas, revealed similar results [62]. This study additionally investigated the dynamics of DNA methylation through different stages of cervical carcinogenesis (that is, normal, invasive and metastatic stages). Hypermethylation at stem-cell PCGT genes was found to occur in cytologically normal cervical cells 3 years before the appearance of the first neoplastic alterations. Moreover, a loss of DNA methylation in CpGs termed ‘methylated embryonic stem-cell loci’ was predominantly observed in invasive tissues, suggesting that hypomethylation at these CpG sites might constitute a poor prognostic signature for these four gynecological tumor entities.

In contrast to these findings, a comparative analysis of methylomes from seven different tissue types revealed that hypermethylated genes tend to be already repressed in precancerous tissues and that aberrant methylation does not contribute to cancer progression under the classical model of epigenetic silencing [63]. It was suggested that pan-cancer patterns of hypermethylation occur owing to the variable gene expression profiles in the corresponding normal tissues. Hypermethylation of specific genes might then account for passenger methylation events rather than for driver events.

Apart from analyzing pan-cancer methylomes, integrative analyses of different tumors harboring mutations in common epigenetic regulators might provide clues about the molecular mechanisms affecting DNA methylation. Guilhamon *et al.* performed an exemplary meta-analysis of the DNA methylation profiles of tumors with *IDH* mutations and intrinsic high methylator phenotypes – namely AML, low-grade GBM, cholangiocarcinomas and chondrosarcomas [64]. The retinoic acid receptor pathway, which is usually dysregulated in the early steps of tumorigenesis, was enriched in the four tumor types. The early B-cell factor 1 (EBF1) was identified as a novel interaction partner of the dioxygenase TET2, suggesting that TET-mediated demethylation is regulated in a tissue-specific manner through EBF1 acting at the transcriptional or post-transcriptional level.

## Clinical applications of DNA methylation in oncology

The identification of a wide number of genes that are affected by aberrant DNA methylation in cancer has highlighted the potential use of this epigenetic modification as a biomarker for cancer risk diagnosis, prognosis and prediction of therapy response. Moreover, the stable nature of DNA compared with RNA and the availability of high-throughput techniques for measurement of DNA methylation in large sample sets add advantages for its clinical application. The most prominent DNA methylation biomarkers are summarized in Table 3.

**Table 3:** **DNA methylation biomarkers and their potential clinical applications**

**Biomarker name**	**Cancer type**	**Tissue detected**
**Risk**		
*BRCA1* DNAm signature (1,829 CpGs)	Breast	Whole blood DNA [65]
140 variable CpGs	Cervical	Normal uterine cervix cells [58]
**Diagnosis**		
*GSTP1*	Prostate	Serum, urine, ejaculate [70]
*APC, EDNRB, GSTP1*	Prostate	Urine [71]
*CDKN2A, ARF, MGMT, GSTP1*	Prostate	Urine [72]
*GSTP1, APC, PTGS2*	Prostate	Paraffin-embedded tissues [110]
*SETP9*	Colorectal	Blood plasma [77]
*APC, MGMT, RASSF2A, WIF1*	Colorectal	Blood plasma [78]
*SHOX2*	NSCLC	Bronchial fluid aspirates/ blood plasma [76]
*CDKN2A, MGMT*	NSCLC	Sputum [74]
*CCND2, RASSF1A, APC, HIN1*	Breast	Fine needle aspiration biopsy [111]
*ZNF154, HOXA9, POU4F2, EOMES*	Bladder	Urine [112]
**Prognosis**		
20-gene signature	ALL	Leukemic cells from bone marrow and peripheral blood [88]
15-gene classifier	AML	
*RASSF1A, APC*	Breast	Serum [82]
*ZAP70*	CLL	CD19 sorted mononuclear cells [80]
*CDKN2A*	CCR	Blood plasma [81]
*DAPK1*	Head and neck	Tumor samples [84]
*DAPK1*	NSCLC	Tumor samples [83]
*CDKN2A, RASSF1A, CDH13, APC*	NSCLC	Primary tumors and lymph nodes [85]
*HIST1H4F, PCDHGB6, NPBWR1, ALX1, HOX9*	NSCLC	Tumor samples [89]
*ALDH1A, OSR2, GATA4, GRIA4, IRX4*	OPSCC	Tumor samples [59]
*GSTP1, APC, PTGS2*	Prostate	Tumor samples [110]
**Response to therapy**		
*BRCA1*	Breast	Tumor samples [92,93]
*BCL2*	Breast	Tumor samples [113]
*PITX2*	Breast	Tumor samples [114]
*TFAP2E*	Colon	Tumor samples [115]
*MGMT*	Glioma	Tumor samples [90,91]
*APAF1*	Melanoma	Tumor samples/cell lines [116]
*IGFBP3*	NSCLC	Tumor samples/cell lines [117]
*BRCA1*	Ovary	Tumor samples [94]

ALL, acute lymphoblastic leukemia; AML, acute myeloid leukemia; CCR, colorectal cancer; CLL, chronic lymphocytic leukemia; DNAm, DNA methylation; NSCLC, non-small-cell lung cancer; OPSCC, oropharyngeal squamous cell carcinoma.

### DNA methylation for risk prediction and as a diagnostic biomarker 

Recently, it has been proposed that the inherent epigenetic variability of normal cells can be used to predict the risk of neoplastic transformation. DNA methylation is being implemented as a molecular biomarker for early cancer detection that is able to distinguish early precancerous lesions from non-cancerous ones. Moreover, the analysis of DNA methylation offers the possibility of non-invasively detecting disease at early stages using biological fluids such as blood, saliva, urine and semen.

For instance, alterations in DNA methylation in healthy cervical tissues collected 3 years before detectable cytological and morphological transformations could predict the risk of acquiring cancer [58]. Differentially variable CpGs showed increased variance in normal cells from people predisposed to cervical neoplasia; the differentially variable CpGs were also enriched for developmental genes and PCGTs. Age-associated variation in DNA methylation was also correlated with the risk of neoplastic transformation.

A study analyzing whole blood from *BRCA1* mutation carriers identified a methylation signature that predicted sporadic breast cancer risk and death years in advance of diagnosis [65]. Hypermethylated CpGs in *BRCA1* mutation carriers were enriched for stem cell PCGTs, demonstrating that alterations of PCGTs occur early in tumorigenesis, as previously described [62,66]. Another study using whole blood samples identified a PCGT methylation signature present in preneoplastic conditions that was prone to become methylated with age, suggesting that age might predispose to tumorigenesis by irreversibly maintaining stem-cell properties [67]. Although attractive as a surrogate tissue, analyses in whole blood should be cautiously interpreted and stringently validated owing to its cellular heterogeneity [68].

Aberrant DNA methylation is also emerging as a potential tool for cancer detection. The list of methylation-based diagnostic biomarkers for different tumor types is enormous. For some of these biomarkers commercially kits are available. Hypermethylation of *GSTP1*, one of the first epigenetic biomarkers to be implemented in the clinic, is used for early diagnosis of prostate cancer [69]. The promoter of this gene is highly methylated in about 90 % of prostate cancers and can be detected in serum, urine and semen [70]. By combining *GSTP1* hypermethylation with (1) the DNA methylation levels of the TSGs *APC* and *EDNRB* [71], (2) the DNA methylation levels of *CDKN2A*, *ARF* and *MGMT* [72], or (3) the levels of the prostate-specific antigen, prostate cancer diagnosis sensitivity is improved [73]. In NSCLC, aberrant DNA methylation of *CDKN2A* and *MGMT* were used to detect malignant lung carcinoma 3 years before its diagnosis using samples from a small cohort of patients [74]. Hypermethylation of the homeobox gene *SHOX2* in bronchial fluid aspirates of more than 500 patient samples allowed the differentiation of benign lung lesions from carcinogenic lesions [75]. A subsequent study analyzing blood plasma from 411 individuals confirmed the specificity and sensitivity of *SHOX2* hypermethylation [76], identifying it as a potential clinical biomarker for early non-invasive lung cancer diagnosis.

Another exemplary diagnostic biomarker is the hypermethylation of SET pseudogene 9 (*SETP9*) in colorectal cancer, which can be sensitively and specifically detected in blood plasma and is able to differentiate between all the stages of the disease [77]. Tumor-specific methylation of *APC*, *MGMT*, *RASSF2A* and *WIF1* have also been suggested as potential biomarkers for early detection of colorectal cancer [78]. Moreover, a recent genome-wide screen using DNA methylation data from more than 700 colorectal cancer samples identified hypermethylation of the thrombin receptor *THBD* and of *C9orf50* as novel blood-based biomarkers for colorectal cancer detection [79].

### DNA methylation as a prognosis biomarker 

In addition to its diagnostic applications, aberrant DNA methylation could help to predict and stratify patients with risks of distinct clinical outcomes. Studies using DNA methylation as a prognostic biomarker have identified more aggressive tumors and predicted overall survival and risk of disease progression and/or recurrence. Initially, studies combined clinical characteristics with aberrant DNA methylation at single or multiple genes, but genome-wide DNA methylation profiling of thousands of CpG sites is now leading to the identification of prognostic signatures.

In CLL, DNA methylation of a single CpG within the zeta-chain-associated protein kinase 70 (*ZAP70*) gene promoter predicted disease outcome better than current genetic approaches [80]. Examples of other hypermethylated genes used to predict poor clinical prognosis include *CDKN2A* in colorectal cancer [81], *RASSF1A* and *APC* in breast cancer [82], the apoptosis-associated gene *DAPK1* in lung and head and neck cancers [83,84], and *CDKN2A*, *RASSF1A*, cadherin 13 (*CDH13*) and *APC* in stage I NSCLC [85].

The first studies characterizing DNA methylation at a genome-wide scale and using large cohorts of patients to investigate prognostic signatures were performed on hematopoietic malignancies. In AML, the methylomes of 344 patients were used to classify 16 distinct AML subgroups. From these, 5 subgroups defined new AML subtypes without any reported cytogenetic, molecular or clinical features. This study also revealed a 15-gene methylation classifier that predicted overall survival [86]. A recent investigation that focused on cytogenetically normal AML patients identified a seven-gene score which combined DNA methylation and gene expression and was associated with patient outcome [87]. In childhood acute lymphoblastic leukemia (ALL), distinct biological ALL subtypes were identified, as well as a group of genes whose DNA methylation levels correlated with a higher risk of relapse [88]. Another study in HPV-driven oropharyngeal squamous cell carcinoma defined a DNA methylation score of five genes (*ALDH1A2*, *OSR2*, *GATA4*, *GRIA4* and *IRX4*), which was associated with clinical outcome [59]. Moreover, DNA hypermethylation of five genes (*HIST1H4F*, *PCDHGB6*, *NPBWR1*, *ALX1* and *HOXA9*) was used to classify high- and low-risk stage I NSCLC and patients with shorter relapse-free survival [89]. Apart from these studies, the efforts of TCGA have shown that methylomes could be used to stratify tumors with distinct biological and clinical characteristics, as mentioned earlier.

### DNA methylation as a biomarker to predict treatment response 

The individual response of each patient to chemotherapeutic drugs is quite heterogeneous and, hence, biomarkers that predict response to therapy as well as the development of drug resistance are urgently required. DNA methylation has proven to be a suitable biomarker to predict treatment outcome in various types of tumors. Such a marker was identified in GBM, where hypermethylation of the DNA repair gene *MGMT* predicted treatment response. Silencing of *MGMT* diminishes DNA repair activity and removal of alkyl lesions, and thus predicts responsiveness to chemotherapeutic agents such as temozolomide and carmustine [90,91]. TCGA confirmed these findings and further identified that *MGMT* hypermethylation in GBM patients might predict responders from non-responders more accurately than the classical expression subgroups [48].

Hypermethylation of the DNA repair gene *BRCA1* in sporadic triple-negative breast tumors has also been proposed as a biomarker to predict sensitivity of breast cancers to the cross-linking agent cisplatin [92] and to the poly(ADP)-ribose polymerase inhibitor olaparib [93]. Similar results were observed in ovarian tumors with *BRCA1/2* mutations, where *BRCA1* hypermethylation predicted better response to poly(ADP)-ribose polymerase inhibitor treatment [94].

### Therapeutic use 

Owing to its reversible nature in comparison to genetic alterations, aberrant DNA methylation can also be therapeutically targeted. Epigenetic drugs such as the histone deacetylase (HDAC) inhibitors, DNA demethylating agents or small molecule inhibitors of the BET family of bromodomain proteins have been shown to modify chromatin structure and modify DNA methylation patterns across the genome [95,96]. DNMT inhibitors can be incorporated into the DNA or RNA of replicating cells, blocking the catalytic domain of DNMTs and thus inhibiting the maintenance of DNA methylation after cell division. The DNMT inhibitors azacitidine (5-azacytidine) and decitabine (5-aza-2'-deoxycytidine) have been tested in clinical trials for hematopoietic malignancies and were approved by the US Food and Drug Administration for the treatment of MDS and AML [97,98]. Moreover, azacitidine in combination with an HDAC inhibitor has been used as a treatment regimen in a phase II clinical trial for solid tumors including NSCLC, breast cancer and colorectal cancer [95,99]. The results obtained for NSCLC showed durable responses and better patient survival, suggesting that combined epigenetic therapy may have clinical benefits for the treatment of this and other solid tumor types.

## Conclusions and future perspectives

The integration of genome-wide DNA methylation profiles with genomic and other omic profiles is just emerging, and further efforts are needed to complete cross-tumor analyses, which will then help us to understand the molecular mechanisms responsible for the epigenetic defects that can result from aberrant DNA methylation. Several interesting findings have been revealed. Subgroups of cancers with high methylation (including CIMP), are associated with individual genomic aberrations underlying these patterns, and have been identified in various cancer entities. At present, however, there is no evidence for a unifying mechanism leading to these high methylation phenotypes.

Moreover, several tumor types, such as basal breast, high-grade serous ovarian and subtypes of serous endometrial, gastric and colorectal carcinomas, related to frequent *TP53* mutations and high levels of SCNAs, share a pattern of low methylation in CGIs. Apparently, in these tumor subtypes, CGIs retain the low methylation patterns observed in normal tissues and are protected from methylation or are subjected to active demethylation. Again, the molecular mechanism underlying these observations is not known. We hypothesize that in this case structural genomic alterations are sufficient to drive carcinogenesis.

Although still in its infancy, pan-cancer methylome analyses have provided some interesting insights into the mechanisms of cancer development. First, it is becoming more apparent that multiple cancer types are affected by mutations in genes encoding epigenetic regulatory enzymes, histone variants and chromatin regulatory factors. Some of these have been experimentally shown to contribute to alterations in methylation patterns. Comparing methylomes across cancer types might now help to identify novel non-recurrent mutations converging on common biological pathways that might lead to the development of altered methylation phenotypes in specific subgroups of cancers. Second, hypermethylation of PCGTs is apparent in basically every tumor type and can even be observed in preneoplastic tissues. Third, the influence of environmental factors on DNA methylomes might have been underestimated until now. For example, infectious agents have been recently linked to specific methylation patterns.

However, pan-cancer methylome analyses still need to overcome some challenges. First, in the past, DNA methylation data were generated on two different platforms for some tumor types. Integration of these data restricts the output to overlapping CpG sites, mostly representing CGIs, and strongly reduces the genome-wide coverage. With the generation of larger datasets derived from the 450 k platform, these limitations will be overcome in the future. Second, comparing datasets derived from different platforms, and from samples provided by various centers, is intrinsically prone to systematic batch effects that need to be carefully monitored. Third, some tumor types are characterized by high tumor heterogeneity that is difficult to control and might lead to false positive results. Also, high tumor purity is an important prerequisite for correct data interpretation, but is often difficult to achieve. Enrichment of certain cell types by sorting or laser capture microdissection prior to analysis might be desirable. Fourth, for the development of clinical predictive, diagnostic or prognostic biomarkers and stratification of patient subgroups, the availability of well documented clinical data is essential. Last, integrative and comparative analyses of multi-platform datasets require powerful bioinformatic and biostatistical algorithms. Dedicated computational centers have to develop and rigorously test and validate these tools.

The epigenetic field is rapidly evolving, and in the near future more single-base resolution methylomes for a large number of tumors will be available. The generation of such methylomes is now affordable due to a considerable reduction in next-generation sequencing costs, improved computational expertise and emerging technologies that use lower DNA input, such as tagmentation-based WGBS. This method is used for WGBS library preparation, and is based on the enzymatic activity of a transposase to simultaneously fragment and tag DNA with adapters [100]. High-resolution methylation maps will provide additional information to the current methylomes, especially regarding cytosine methylation in a non-CpG context, long-range methylation interactions, and better assessment of allele-specific DNA methylation (reviewed in [101]). In addition, high sequencing coverage will accurately quantify DNA methylation in genomic regions such as enhancers, insulators, intergenic regions and repetitive elements, which are currently not included in pan-cancer methylome analyses.

In the longer term, novel technologies will also allow genomic and epigenomic analyses of single cells. These analyses will generate more precise datasets by avoiding the problems associated with tissue impurities or heterogeneity, and will allow a direct link between the methylome and the transcriptome [102]. However, the broad application of single-cell analyses still requires methodological development to reduce technical artefacts. To fully understand the interplay between the genome, epigenome and transcriptome, existing datasets need to be integrated with information about additional mechanisms of epigenomic regulation, including the emerging non-coding transcriptome and higher-order chromatin organization. Importantly, hypotheses generated from these combined efforts need to be experimentally tested to prove their functional relevance.

Finally, in terms of translation to the clinic, an essential aspect is to use the knowledge generated by methylome analyses as well as from the integration of methylation data with other omic data to identify novel clinical markers that should be able to stratify patients better and to define molecular signatures across different tumor types. On the basis of these molecular markers, novel epigenetic therapies could be developed, setting the stage for better clinical trial strategies across cancer types as well as for personalized medicine based on next-generation sequencing data. Already, pan-cancer analyses have revealed molecular similarities that will allow existing therapies to be applied to different cancer types.

### Box 1 The International Cancer Genome Consortium: characterizing cancer genomes in different tumor types

Cancer genomes are complex. The integration of comprehensive catalogues of genomic, transcriptomic, epigenomic and proteomic data is a promising strategy for tackling this complexity. Institutions from across the globe have joined forces to achieve this ambitious goal. In 2006, The Cancer Genome Atlas (TCGA) Research Network was launched in the USA with the aim of generating molecular profiles of thousands of samples from more than 25 distinct tumor types [2]. A year later, the International Cancer Genome Consortium (ICGC) was created, with the goal of characterizing genomes from 50 different cancer types and subtypes worldwide [103]. By 2013, TCGA – now an ICGC member – produced comprehensive molecular profiles of more than 7,000 samples from 27 types of cancer [2]. All the data generated by these research networks are publicly available via the ICGC [104], TCGA [105] and the cancer genomics hub [106] data portals.

To make these data comparable, the ICGC aims to standardize the collection, processing and analysis of samples across multiple institutions. Infinium HumanMethylation27 and HumanMethylation450 BeadChips have been used by ICGC to produce genome-wide DNA methylation profiles. From at least 15 cancer methylomes generated so far, the breast cancer methylome comprises the largest number of samples, followed by serous ovarian and kidney renal clear cell carcinoma (Table 1). Moreover, whole-genome bisulfite sequencing (WGBS) will be applied for some tumors and has already been used to generate the methylomes of pediatric brain tumors and chronic lymphocytic leukemia (CLL).

## Abbreviations

2-HG: d-2-hydroxyglutarate
5hmC: 5-hydroxymethylcytosine
5mC: 5-methylcytosine
ALL: acute lymphoblastic leukemia
AML: acute myeloid leukemia
CGI: CpG island
CIMP: CpG island methylator phenotype
CLL: chronic lymphocytic leukemia
DMV: DNA methylation valley
DNMT: DNA methyltransferase
EBF1: early B-cell factor 1
EBV: Epstein-Barr virus
ER: estrogen receptor
GBM: glioblastoma multiforme
HDCA: histone deacetylase
HPV: human papilloma virus
ICGC: International Cancer Genome Consortium
MDS: myelodysplastic syndrome
MSI: microsatellite instability
NSCLC: non-small-cell lung carcinoma
PCGT: polycomb group target
PMD: partially methylated domain
PR: progesterone receptor
PRC: polycomb repressor complex
SCNA: somatic copy-number alteration
SQCC: squamous cell carcinoma
TCGA: The Cancer Genome Atlas
TET: ten-eleven translocation
TSG: tumor suppressor gene
WGBS: whole-genome bisulfite sequencing
